# Radical Gastrectomy: Still the Cornerstone of Curative Treatment for Gastric Cancer in the Perioperative Chemotherapy Era—A Single Institute Experience over a Decade

**DOI:** 10.1155/2018/9371492

**Published:** 2018-01-14

**Authors:** Harsh Kanhere, Raghav Goel, Ben Finlay, Markus Trochsler, Guy Maddern

**Affiliations:** ^1^University of Adelaide Discipline of Surgery, The Queen Elizabeth Hospital, Woodville, Adelaide, SA, Australia; ^2^Division of Surgery, The Queen Elizabeth Hospital, Woodville, Adelaide, SA, Australia

## Abstract

**Background and Objectives:**

Most gastric cancer patients now undergo perioperative chemotherapy (POCT) based on the MAGIC trial results. POCT consists of neoadjuvant chemotherapy (NACT) as well as postoperative adjuvant chemotherapy. This study assessed the applicability of perioperative chemotherapy and the impact of radical gastrectomy encompassing a detailed lymph-node resection on outcomes of gastric cancer.

**Methods:**

Medical and pathology records of all gastric carcinoma resections were reviewed from 2006 onwards. Pathological details, number of lymph-nodes resected, and proportion of involved nodes, reasons for nonadministration of NACT, complications, recurrence, and survival data were analysed.

**Results:**

Only twenty-eight (37.8%) out of 74 patients underwent NACT and only nine completed POCT. NACT was declined due to comorbidities/patient refusal *n* = 24, early stage *n* = 14, and emergency presentation *n* = 8. Patients receiving NACT were much younger. Anastomotic leaks, hospital-mortality, lymph-node yield, and proportion of involved lymph-nodes were similar in both groups. Thirty-two patients died due to recurrence with lymph-node involvement heralding higher recurrence risk and much poorer survival (HR 2.66; *p* = 0.013).

**Conclusion:**

More than 60% patients with resectable gastric carcinoma did not undergo NACT. Radical gastrectomy with lymphadenectomy remained the cornerstone of treatment in this period.

## 1. Introduction

Gastric carcinoma remains a leading cause of cancer related death [1]. Although there is a trend towards improved survival in the last decades, the survival rates remain low at 5 years [2]. Since the publication of the MAGIC [3] trial which showed a 13% improvement in 5-year survival with perioperative chemotherapy (POCT) (36% versus 23%), most units have adopted this approach in treatment of advanced stage gastric cancers.

Randomised trials provide the highest level of evidence in patient management. Adoption and implementation of the trial protocols in daily practice however are not always easy. It is important to assess the feasibility, adoption, implementation, and benefits achieved from change of practice based on trial results in day to day practice to gauge the true impact on patient management.

This study was undertaken to assess the outcomes of all resectable gastric cancers that presented to a tertiary referral centre in South Australia. The aim was to identify the applicability of the POCT protocol, pattern of treatment, the pathological features, and the clinical outcomes in this cohort over a period of 10 years. The reasons for patients not undergoing neoadjuvant chemotherapy (NACT)/POCT were identified and factors impacting the oncological outcomes were analysed. The use of POCT in all patients with resectable gastric cancers was scrutinised.

## 2. Materials and Methods

This study was approved by the Human Research Ethics Committee of The Queen Elizabeth Hospital as a part of the audit process of an existing database.

A prospective database for all patients with upper gastrointestinal cancer resections has been maintained at our institute from 2005. POCT protocol for gastric cancers based on the MAGIC trial results [3] was adopted at the hospital in 2006. This study was a retrospective analysis of all gastric cancer resections from then until June 2016. Patient data such as age, gender, presence of significant comorbidities, date of diagnosis, and surgery were recorded. All surgeries were performed with an open approach. The authors believe this is the best approach in their hands with an oncological perspective. Surgeons performing gastrectomy are skilled in minimal invasive and laparoscopic surgery and utilise this approach regularly for benign upper GI pathologies as well as complex bariatric surgeries. All patients with stages T2 or N1 disease were considered for NACT. A standard subtotal/total gastrectomy in conjunction with a lymphadenectomy was performed. Routine lymphadenectomy involved Level 1–12 clearance. Level 10 clearance (splenic hilar nodes) was performed selectively. Lymph-node resections from levels 1–6 were performed based on site of the primary disease. Lymphadenectomy was less radical in some settings such as emergency cases or when the procedure was performed with more of a palliative intent or in patients with borderline fitness. In terms of fitness, the patients were evaluated initially at the surgical review with regard to number of comorbidities and their respective severity. Accordingly they were referred to high-risk preoperative clinic for further evaluation. Patients underwent an echocardiogram and pulmonary function tests as part of the preassessment for NACT. The decision for NACT was ultimately taken by the medical oncology team based on their assessment and MDT evaluation. All patients underwent an endoscopy, staging CT of chest, abdomen, and pelvis as well as a laparoscopy prior to NACT. These investigations were repeated in the interval between NACT and surgery with EUS performed in selected cases. The preoperative staging, site of disease (proximal versus distal), type of surgery, intra- and postoperative complications, and detailed histopathology results were documented. Case records of all patients were retrieved and data regarding neoadjuvant and postoperative treatment was collected. Reasons for not undergoing NACT/POCT were documented in patients who underwent surgical resection only.

Every case was discussed in both an institute based meeting and statewide Multidisciplinary Team Meeting (MDT). Decision to proceed to NACT or directly to surgery was based on the recommendations of the MDTs. A study to look at the concordance between the two meetings is in the pipeline. In the instances of disagreement, the decisions from both meetings were discussed with the patient with precedence given to the statewide MDT decision.

### 2.1. Statistics

All continuous data was compared using a paired *T*-test while categorical data was expressed in proportions and compared using Fischer's exact test and Chi2 test where appropriate. Survival was presented as Kaplan-Meier curves and assessed using Cox proportional hazards model. Time to recurrence was presented as cumulative incidence function curves and assessed using Fine and Gray competing risk regression models.

## 3. Results

### 3.1. Sample Characteristics

A total of 74 patients underwent surgical resection for gastric carcinoma during the study period. Of these only 28 (37.8%) were deemed eligible as per MDT recommendation and received NACT prior to surgery. Mean age was 67.9 years (32–87 years). Patients undergoing surgery alone were significantly older than those that underwent NACT and surgery (mean age 69.9 versus 64.1 years; *p* = 0.04). Patients deemed ineligible for chemotherapy based on comorbidities were even older with a mean age of 76.6 years (65–86 years; *p* = 0.0002). Comorbidities included cardiac failure or cardiomyopathy precluding platin based chemotherapy. The sample characteristics and reasons for not receiving NACT are summarised in Table 1. Associated comorbidities and age were the most common reasons for declining NACT (24/46 patients). Prevalent comorbidities included cardiac failure/ischaemic heart disease (20%), severe COPD (15%), and multiple medical problems (10%). Fourteen patients were declined because of presumed early stage disease on preoperative staging and eight due to emergency presentation of obstruction or bleeding. There were 53 distal and 21 proximal cancers in the patient cohort. A high proportion of patients with proximal cancers underwent NACT (15/21). All 21 patients with proximal cancers underwent a radical total gastrectomy while a subtotal radical gastrectomy was performed in those with distal cancers. A median of 16 (3–49) lymph-nodes were resected per patient. The number of lymph-nodes resected were similar in patients undergoing NACT and surgery and those undergoing surgery alone (19 versus 15; *p* = 0.053). Of the 74 resections, 45 had a lymph-node yield of more than 15 nodes and 15 of these were more than 25 nodes. The proportion of positive lymph-nodes was also similar in the two groups (2.15 versus 1.9; *p* = 0.7). Of the 28 patients in the NACT group, 17 had positive lymph-nodes on histology. Only 9/28 (32%) patients went on to complete the postoperative arm of the chemotherapy in the POCT group. Of the 23 patients with node positive disease in the no NACT arm, seven (30.4%) went on to receive postoperative chemoradiotherapy. Only two patients deemed early stage showed positive lymph-nodes on final histology. Further details are illustrated in Table 2.

### 3.2. Morbidity

Significant morbidity due to chemotherapy occurred in 5 (17.8%) patients (4 DVT, 1 febrile neutropenia). Postoperative morbidity (Clavien-Dindo grade 3 and above) and anastomotic leaks were similar in both groups as shown in Table 2. There were 2 (2.7%) postoperative mortalities within 30 days. One patient died on postoperative day 1 with small bowel ischemia, considered to be a vascular event (with no evidence of internal herniation/strangulation at reexploration), and the other had a myocardial infarct. Both occurred in patients not receiving neoadjuvant chemotherapy with significant comorbidities.

### 3.3. Recurrence and Survival

**Figure 1:**
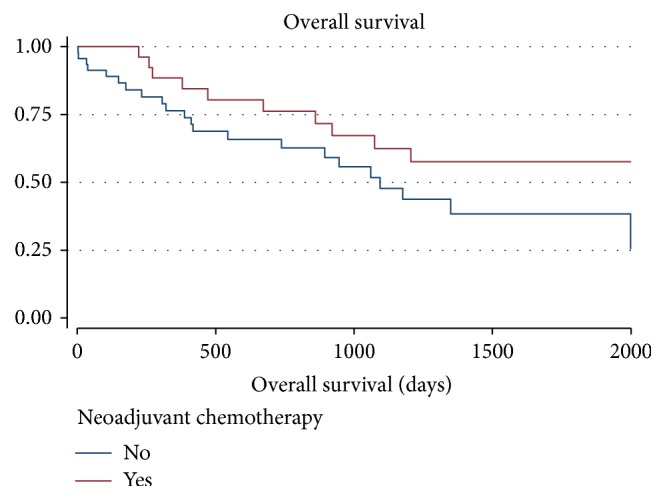
NACT versus no NACT HR − 1.00 no NACT; 0.56 NACT; 95% CI 0.26–1.18.* p* = 0.128.

**Figure 2:**
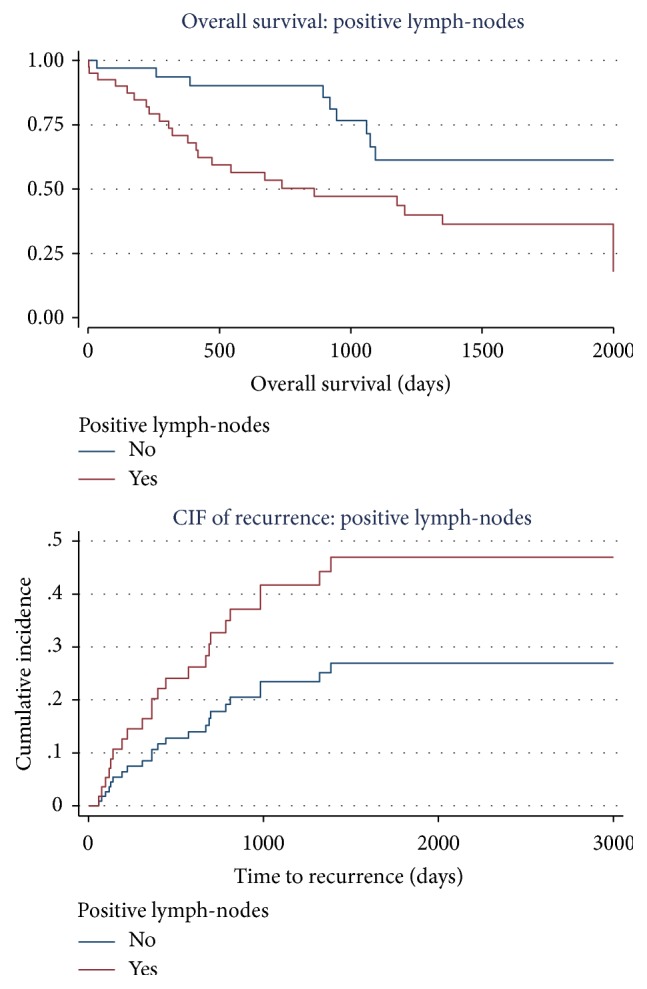
(a) Overall survival: positive lymph-node versus negative lymph-node status. (b) Risk of recurrence: positive lymph-node versus negative lymph-node status.

Thirty-two (43.2%) patients died of disease during the observation period. Amongst the patients who died (10 NACT, 22 no NACT), overall survival ranged from 0 to 65.7 months with a mean of 19.2 months and median of 13.6 months. Median survival was significantly poorer in patients with positive lymph-nodes than those without (17.1 versus 24.3 months, HR 2.66; *p* = 0.013). Survival was slightly better in patients undergoing neoadjuvant chemotherapy but did not reach statistical significance (20.8 versus 19.0 months *p* = 0.128). Survival curves and time to recurrence cumulative incidence function curves are depicted in Figures 1 and 2.

## 4. Discussion

This study details the 10-year experience with perioperative chemotherapy in gastric carcinoma at a tertiary centre in South Australia. The protocol for perioperative chemotherapy was introduced at the hospital after the publication of the results of the MAGIC trial [3]. The improvement in 5-year survival with perioperative chemotherapy was significant in this trial even with a major proportion of patients not completing the postoperative treatment.

Significant advantages seen in the trial setting however may not be evident in wider community based clinical practice for various reasons, especially if the treatment cannot be provided to all patients. According to Post et al. [4] randomised controlled trials (RCTs) are the preferred source of evidence for the effect of treatment but patients participating in RCTs often manifest important differences from patients seen in clinical practice. These differences may be in the form of age, comorbidities, and type of presentation, which will have an important bearing on the outcomes.

### 4.1. Eligibility and Uptake of POCT

Just over a third (37%) of all patients presenting with resectable gastric cancers were deemed eligible for perioperative chemotherapy after thorough evaluation by the Surgical and Medical Oncology teams. All cases were discussed in an institute based MDT as well as a statewide MDT and treatment decisions were made on the basis of consensus opinion at both meetings. The most common reason for not receiving chemotherapy preoperatively was comorbidities precluding multimodality treatment (*n* = 24; 32.4%). Emergency presentations with gastric outlet obstruction and/or bleeding were the other main reasons to proceed directly to surgery. These results are consistent with many studies [5]. A large study based on American College of Surgeons data showed a similar uptake of NACT (36.6%) with large academic centres using NACT more frequently [6]. Shrikhande et al. [7] reported that up to 40% patients did not receive NACT due to emergency presentation or early stage disease. It is the authors' belief that this data reflects the real clinical practice situation in most countries and centres.

Only nine of the 28 patients (32.1%) went on to complete the postoperative chemotherapy arm in this study. Others have shown that more than 60% of patients who undergo NACT are unable to complete the postoperative treatment [5]. The role of the postoperative part of the chemotherapy is thus questionable. The MAGIC trial itself showed survival benefit despite majority of the patients in the chemotherapy arm not completing the postoperative component of the chemotherapy. The authors believe that further trials or meta-analyses are required to test the benefit/risk ration and effectiveness of this part of the regimen.

The patients who did not receive NACT were significantly older than the NACT patients (69.9 versus 64.0 years). The average age of the whole cohort was older than the MAGIC trial (67.7 versus 62 years). Most studies show a similar pattern [7]. A larger proportion of proximal gastric cancers were treated with NACT than upfront surgery (15/21). Similar results are reported by other authors [8]. The perception that proximal gastric cancers present at a later stage and consequently have a poorer prognosis [9] may be responsible for this phenomenon.

### 4.2. Lymphadenectomy

The role of lymphadenectomy cannot be understated. A modified D2 gastrectomy is the accepted norm in today's practice [10]. Almost all patients in this study cohort undergoing elective surgery underwent a modified spleen preserving D2 lymphadenectomy. The number of nodes retrieved is particularly relevant in gastric cancer resections. A recent study shows that retrieving more than 25 lymph-nodes during curative-intent gastrectomy substantially improved survival of advanced gastric cancer without compromising patient safety [11]. Previous studies have shown that retrieval of 15 lymph-nodes constitutes an adequate lymphadenectomy [12]. In stage II and III disease, removal of >15 LN appears to contribute to a considerable survival advantage [12, 13]. An extended lymphadenectomy will most reliably allow >15 LN to be removed and adds no operative morbidity and mortality according to Li et al. [8]. They strongly recommend such a lymphadenectomy in curative resections of gastric cancer. A D2 lymphadenectomy thus provides vital information in prognostication of the disease and affords survival benefit as well [11, 13].

It is important to note that the number of lymph-nodes harvested was not affected by NACT (20.6 NACT group versus 15.8 no NACT). Some studies have indicated that NACT reduces the number of lymph-nodes harvested as compared to upfront surgery. Wu et al. [14] found a reduced lymph-node yield (<15 harvested lymph-nodes) following NACT compared to patients who underwent upfront surgery for gastric cancer (7.7% versus 24.1%); however other large series dispute this observation [15].

Seventeen of the 28 patients (60%) had lymph-node metastases on final histology after NACT. Similar data is reported by other authors [7]. This underscores the importance of a good lymph-node clearance even with NACT. It also highlights the fact that NACT may not produce a significant response in lymph-node metastases.

### 4.3. Morbidity and Mortality

There was no difference in the postoperative morbidity or mortality in patients undergoing NACT as compared to those undergoing upfront surgery. NACT is regarded safe in relation to postoperative morbidity and mortality based on the MAGIC trial [3] and recently published data [8]. Some authors have reported an increase in wound infection rates and duodenal stump insufficiency in the NACT group [16]. Such issues were not evident in this study. Chemotherapy related DVT/PE was however noted in 4/28 (14.2%) patients in the NACT group. This constituted the significant morbidity from chemotherapy but did not affect the surgical outcomes. Neoadjuvant chemotherapy has been shown to be an independent risk factor for DVT in oesophageal and gastric cancers in a recent publication which recommends pharmacological and mechanical prophylaxis to be commenced in the neoadjuvant period [17, 18]. Targeted and considered use of NACT would be very useful in this regard.

### 4.4. Survival

Survival was only slightly better in the NACT group as compared to the no NACT group (20.8 versus 19.0 months). This may be due to the fact that the two groups were not exactly comparable. There were 14 early stage cancers in the no NACT group, but patients in this group were older and had more comorbidity as well. A recent meta-analysis has suggested that neoadjuvant chemotherapy improves survival in patients with gastric and gastro-oesophageal junctional cancers [19]. Given that all patients are not eligible for NACT, these results are not applicable to all patients. Furthermore, a meta-analysis of 6 randomised trials (781 patients) failed to find any benefit in survival specifically in gastric cancer patients with NACT as compared to surgery alone [20]. It appears therefore that evidence for NACT is robust when gastric, gastro-oesophageal junctional, and distal oesophageal cancers are grouped together as was the case in the MAGIC trial as well as a French trial in perioperative chemotherapy [21]. The authors therefore believe that further investigation is warranted for the use of NACT purely in gastric cancers where the benefit may not be as significant. Cost benefit analyses should also be included in such investigative trials and “value for expense” taken into account while treating gastric cancers with NACT [22].

The authors believe that NACT and indeed multimodality therapy need to be tailored and individualised for gastric cancers. Recent advances in biomarkers predicting chemotherapy response (HER2, P53) show promising results and will be useful to tailor NACT for gastric carcinoma based on the profile of each patient [23]. Newer biomarkers like angiopoietin-2 show promising results for targeted therapy in gastric cancers as well [24]. These approaches are perhaps useful in providing low toxicity targeted treatment that can be tolerated by elderly individuals and those with significant comorbidities instead of NACT and improve survival in this group as well.

### 4.5. Limitations

Being a retrospective analysis, this study has inherent limitations. The final histology of all patients who underwent NACT was not reviewed to assess the tumor response. It is however reported that on the whole only up to 50% gastric carcinomas show tumor response (partial or complete) with NACT [25]. This highlights the fact that 50% of gastric cancers do not show any response to NACT and hence makes a further argument for a targeted approach to NACT. In our cohort, clinical progression was seen in only 2 patients on NACT, but both underwent surgical resections and no metastatic disease was evident. Since it was a retrospective review of patients undergoing surgery till date, 5-year survival is difficult to calculate. The authors acknowledge the fact that most trials of perioperative chemotherapy exclude emergency presentations and early- stage disease. Thus, the denominator of patients eligible for NACT may be 74 − 14 − 8 = 52. It is however our desire to show the real world experience, but even considering the eligibility for most trials based on presentation and stage, close to 50% (24/52), did not receive NACT.

### 4.6. Conclusion

Only about 40% of patients presenting with resectable locally advanced gastric cancers can undergo NACT. A majority of patients will be treated with surgery alone due to various reasons. Targeted low toxicity therapy should be investigated further to improve survival from gastric cancers in all patients. While a proportion of patients benefit from neoadjuvant chemotherapy, radical gastrectomy remains the cornerstone for treatment of gastric carcinoma and an appropriate lymphadenectomy is advocated for prognostic and treatment purposes.

## Figures and Tables

**Table 1:** Sample characteristics.

Sex	
Males	42 (56.7%)
Females	32 (43.2%)
Site	
Proximal	21 (28.8%)
Distal	52 (71.2%)
Neoadjuvant chemotherapy	
No	46 (62.2%)
Yes	28 (37.8%)
Reason for not having NACT (*n* = 46)	
Comorbidities	24 (52.2%)
Early stage	14 (30.4%)
Emergency presentation	8 (17.4%)

NACT: neoadjuvant chemotherapy.

**Table 2:** Pathology and morbidity characteristics.

	NACT *n* = 28	No NACT *n* = 46	*p*
Age [mean, (range)]	64.1 (47–76 years)	69.9 (32–86)	0.04^*∗*^

Proximal/distal	15/13	6/40	0.0004^*∗*^

Number of lymph-nodes harvested [mean, (range)]	20.6 (7–49)	15.8 (3–36)	0.053

Number of positive lymph-nodes [mean, (range)]	2.15 (0–13)	1.90 (0–10)	0.72

Patients with positive lymph-nodes [*n*, % total]	17 (60.7%)	23 (50%)	0.47

Patients with anastomotic leak [*n*, % total]	1 (3.5%)	2 (4.3%)	1.00

Proportion of patients with postoperative complications (%)	28.5%	34.7%	0.23

NACT: neoadjuvant chemotherapy; ^*∗*^statistically significant (*p* < 0.05).

## Additional Points

*Synopsis of Table of Contents.* This study looked at the eligibility of administering perioperative chemotherapy for all resectable gastric cancer patients in a clinical setting. Reasons for ineligibility and problems with administering NACT to all patients are highlighted. Modified D2 lymphadenectomy is important for prognostication and surgical clearance.
